# Flood Disaster Risk Assessment of Rural Housings — A Case Study of Kouqian Town in China

**DOI:** 10.3390/ijerph110403787

**Published:** 2014-04-03

**Authors:** Qi Zhang, Jiquan Zhang, Liupeng Jiang, Xingpeng Liu, Zhijun Tong

**Affiliations:** School of Environment, Northeast Normal University, Changchun 130024, China; E-Mails: zhangq941@nenu.edu.cn (Q.Z.); jack860614@sina.com (L.J.); liuxp912@nenu.edu.cn (X.L.); gis@nenu.edu.cn (Z.T.)

**Keywords:** flood disaster, risk assessment, rural housings, “3S” technology

## Abstract

Floods are a devastating kind of natural disaster. About half of the population in China lives in rural areas. Therefore, it is necessary to assess the flood disaster risk of rural housings. The results are valuable for guiding the rescue and relief goods layout. In this study, we take the severe flood disaster that happened at Kouqian Town in Jilin, China in 2010 as an example to build an risk assessment system for flood disaster on rural housings. Based on the theory of natural disaster risk formation and “3S” technology (remote sensing, geography information systems and global positioning systems), taking the rural housing as the bearing body, we assess the flood disaster risk from three aspects: hazard, exposure and vulnerability. The hazard presented as the flood submerging range and depth. The exposure presented as the values of the housing and the property in it. The vulnerability presented as the relationship between the losses caused by flood and flood depth. We validate the model by the field survey after the flood disaster. The risk assessment results highly coincide with the field survey losses. This model can be used to assess the risk of other flood events in this area.

## 1. Introduction

There is no doubt that global climate change has increased the frequency of extreme precipitation events and may cause many more floods. This brings great human life and property losses by flooding fields, washing away housings, infectious diseases, *etc*. China is a developing country with a large population. About half of the population lives in rural areas. Previous studies have mainly focused on the influences of flood disasters on cities and agriculture, and there are seldom studies on the impacts of flood disasters on rural houses, which are much more vulnerable to disasters because of the weak building structure, and lack of sufficient attention in disaster prevention and mitigation. Therefore, it is very necessary to assess the flood disaster risk of rural housing.

According to the statistics, the flood disaster that happened in Jilin Province in 2010 caused a total of 118,000 houses to collapse and damaged 301,000 houses, among which rural housing accounted for 75% or even over 95% in some areas, Kouqian Town suffered an even larger flood with a return period of 1,600 years. As mentioned, flood disaster risk assessment studies are mainly focused on the cities, such as Yin *et al.* who assessed the waterlogging disaster risk of the Jing’an District in Shanghai by creating a GIS raster-based urban water-logging model to simulate the water depth and inundation areas, using vulnerability curves to evaluate the possible losses [1]. Chen *et al.* took downtown Ha-Erbin (Daoli District) as an example to study the trip difficulty for urban residents in various depths of water after urban rainstorm waterlogging, and scenario simulation was used to build the waterlogging model [2]. Studies of flood disaster risk for rural areas mainly focus on agriculture, and there are seldom studies for rural housing. Xu used VR-GIS technology to determine flood inundation areas and establish an emergency decision-making system during the flood of a small town [3,4]. Yin *et al.*, and Liu *et al*. analyzed the flood pressure and the flood resistance of rural housing under different flood hydraulic and wave conditions by physics experiments and models, and a flood resistance capacity evaluation system of rural housing was developed based on this research [5,6]. Flood disaster risk is the combination of hazard, exposure and vulnerability [7]. These studies only focus on the hazard of flood disaster or the resilience characteristics of the rural housing stock.

The definition of risk varies with the applications for which they are used and it can be seen graphically as a function of these three elements, hazard, exposure and vulnerability as illustrated in the risk triangle proposed by Crichton [8,9]. In this study, flood risk was assessed from hazard, exposure and vulnerability by using “3S” techniques combined with field sampling survey and a new flood risk assessment method for rural housings was proposed. The results would help the management of flood disasters, and be valuable for guiding the rescue and relief goods layout, and reducing the losses as much as possible.

## 2. Study Area

**Figure 1:**
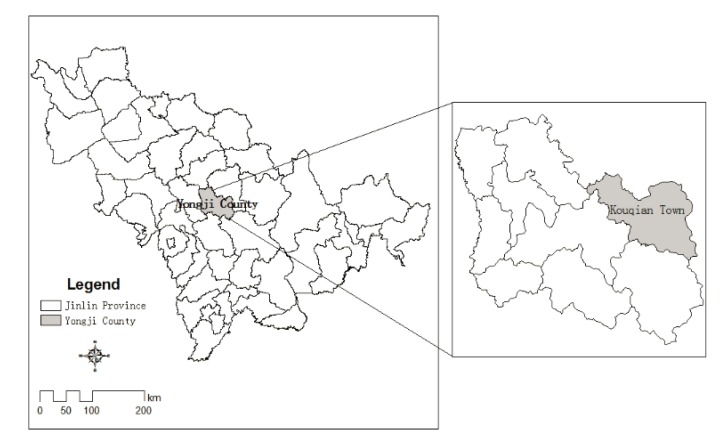
The location of the study area.

Kouqian Town is located in the northeastern of Yongji city of Jilin Province (Figure 1), with an area of 349.22 km^2^. There are seven communities and fifteen administrative villages in Kouqian Town, and the total population is 37,621 households. Kouqian Town is located in the transition zone of the Songnen plain to the Changbai Mountain, and the Wuli River and Sijian River join together in the north of Kouqian Town. The town is located in the northeast monsoon region, with hot, rainy summers and cold, dry winters. Precipitation is concentrated in summer. More than 10 serious flood events have happened in this area since 1950, such as in the years 1951, 1953, 1986 and 2010. They caused many houses to be washed away, casualties, and property losses.

## 3. Data and Methods

### 3.1. Research Methods

#### 3.1.1. Remote Sensing Image Interpretation Method

Remote sensing image interpretation is the process of obtaining target object information from remote sensing images. There are many methods of remote sensing interpretation used to identify flood range. This study based on the characteristics of the study area and the parameters characteristic of the remote sensing image, choosing normalized differential water index (NDWI) [10], the combination of density segmentation and visual interpretation to interpret the flood submerged area.

#### 3.1.2. Grid GIS Method

Grid GIS method is the inheritance and development of grid map taking the information grid as the basic study unit, which is different with traditional administrative boundaries basis data collection and editing. In this study, the methods of spatial interpolation, remote sensing retrieval and multivariate analysis were used to distribute the data into each grid cell.

#### 3.1.3. Fieldwork Data Collection

The fieldwork activities consisted of three major processes: indoor preparation, interviews with households and data processing. During indoor preparation, relevant data and information about the study area were collected to develop a detailed plan. In the second stage, GPS was used to locate each sampling point, and data was obtained by actual measurement and questionnaire. In the final data processing stage, fieldwork data was classified to establish the database.

### 3.2. Data Requirement

The remote sensing image data was obtained from the Chinese Resources Satellite Application Center and Google Earth, the sensing image taken by CCD sensor on Environmental disaster mitigation satellite. Terrain data was gained from 1:10,000 scale topographic maps made by the National Mapping Office. Meteorological data was gained from the Meteorological Data Sharing Service of China. The number of households in each community was obtained from the government of Kouqian Town. The flood disaster housing losses were determined using field surveys.

### 3.3. Flow Chart of Study

**Figure 2:**
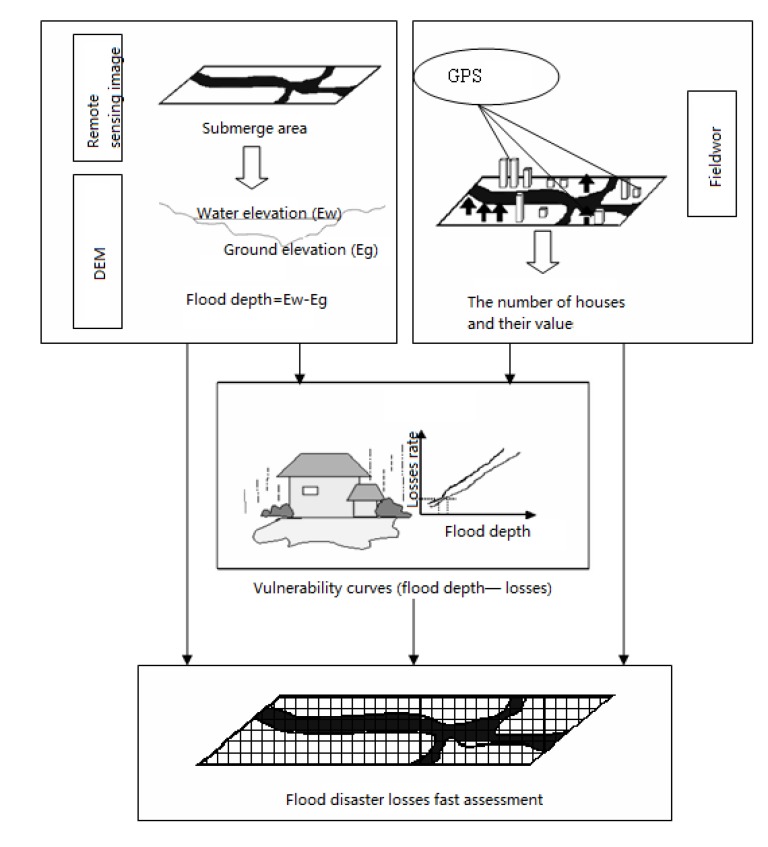
Flow chart.

This research extracted the flood submerged area and flood depth by using the remote sensing images of the flood and DEM data. Based on the conceptual framework of housing exposure the assessment model for the value of housing structure and indoor property was built, and the property distributed in grids by using grid GIS technology. The flood depth and losses regression models were built for housing structures and indoor property based on the fieldwork data. Finally, we got the flood disaster losses fast assessment model. The flow chart is seen in Figure 2.

## 4. Results and Discussion

### 4.1. Grid Division

The division of the grid includes the definition of grid size and determination of the shape. Determine the grid size must guarantee the accuracy and efficiency of flood depth calculation, and take into account the size and spatial distribution of disaster bearing body. The determination of grid shape is mainly according to the spatial pattern of the disaster bearing body. The bearing body of this study is mainly housing, so the shape of the grid is rectangular. The grid size was defined as 80 m by analyzing the high-resolution remote sensing images of Kouqian Town and measuring the size of houses in Kouqian Town.

**Figure 3:**
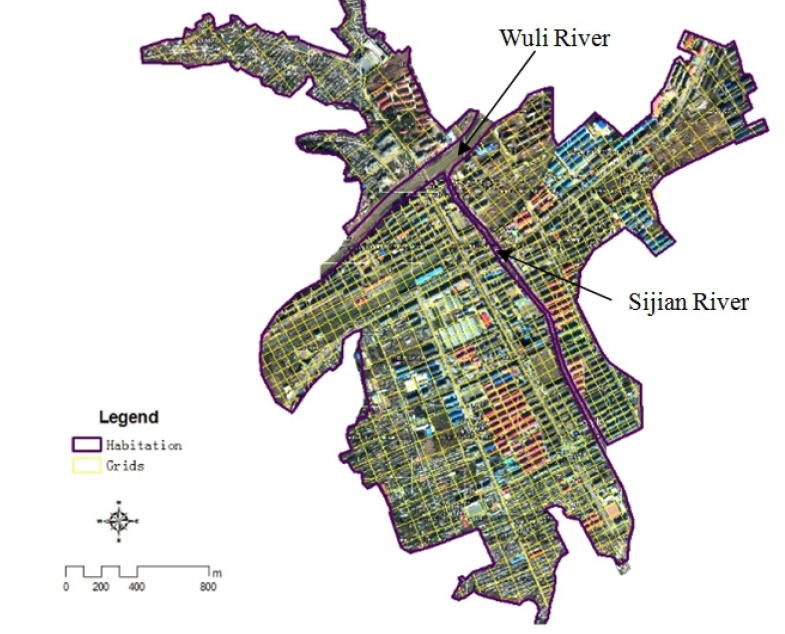
The result of grid division in Kouqian Town.

Rectangular grids were generated by using the HawthsTools of arcGIS software, and the length of grid edge is 80 m. Adjusting the auto-generated grid cells by comparing the high-resolution remote sensing image to make sure one house will not be divided into different grid cells, and combining the small cells at the edge. Finally, we got the grid cells of Kouqian Town (Figure 3).

### 4.2. The Hazard of Flood Disaster Risk

The hazard of flood disaster means the intensity and frequency of flood threatened areas be affected by the flood. It is common to use submerged area, flood depth, duration and frequency to portray this. Flood submerged area and flood depth are the key factors that affect flood disaster risk and residential losses. In this study, flood submerged area and flood depth were selected as the assessment factors of hazard of flood disaster. Flood submerged area was identified from remote sensing images by using NDWI and density segmentation methods. Flood depth was calculated by using the Voronoi Map and Grid GIS methods.

#### 4.2.1. Remote Sensing Interpretation of Flood Submerging Range

The main feature type in the study area includes the water body (including the original water and flood water), vegetation and residential areas. Using the 4, 3, 2 band of environmental disaster reduction satellite CCD data to make standard false color composite, the interpretation keys was established through field investigation using GPS to locate the typical objects of the study area combined with the corresponding points of the image features for visual interpretation (Table 1). Flood water would be identified through interpretation keys from the remote sensing images.

**Table 1:** Interpretation keys of main landmarks in Kouqian Town.

Image	Interpretation Key	Number	Landmark
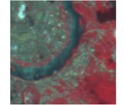	The color of the river is dark blue or blue. It was natural bent just like banded and usually has branches.	1	River
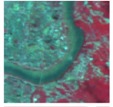	The flood water was dark on the image, and it is slightly yellowish compared to the rivers, lakes and other natural water bodies.	2	Flood
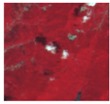	Vegetation is red or dark red and its image structure is more uniform.	3	Vegetation
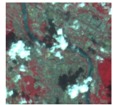	Residential area has a platy texture and the river often passes through. Farmland is widely distributed around residential area which is surrounding by developed traffic.	4	Habitation

**Figure 4:**
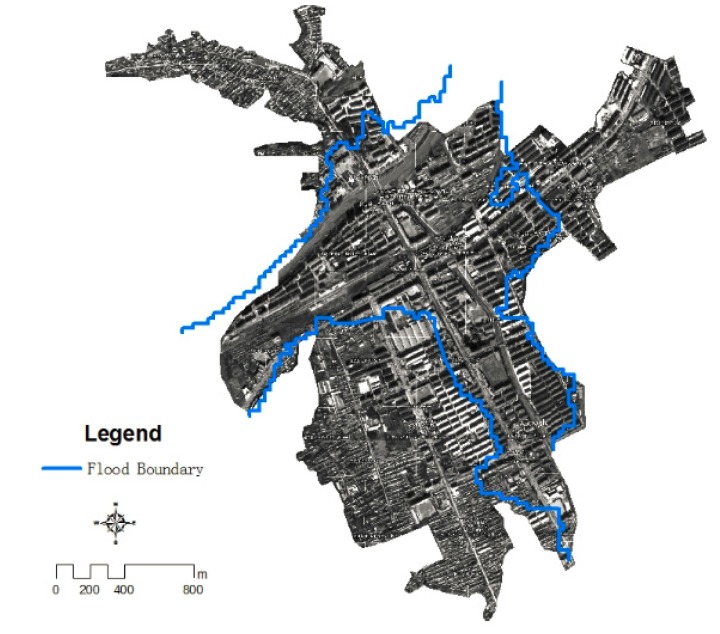
Results of flood submerging range of study area.

Because vegetation has a unique reflection peak in the near infrared, vegetation had been extracted from remote sensing images by band 2 and 4 of satellite CCD data using the NDWI method based on ENVI software and contrast standard false-color image to set the threshold value [10]. The classification results were converted to vector data for boundaries in order to cut near-infrared bands of satellite remote sensing images by using ARCGIS software, and the submerged area of the flood was extracted by using density segmentation method based on ENVI software. The flood submerged area was transformed into vector data and put it into ARCGIS, the results of the scope of flood had been obtained by removing the spots of noise (Figure 4).

#### 4.2.2. The Calculation of Flood Depth

**Figure 5:**
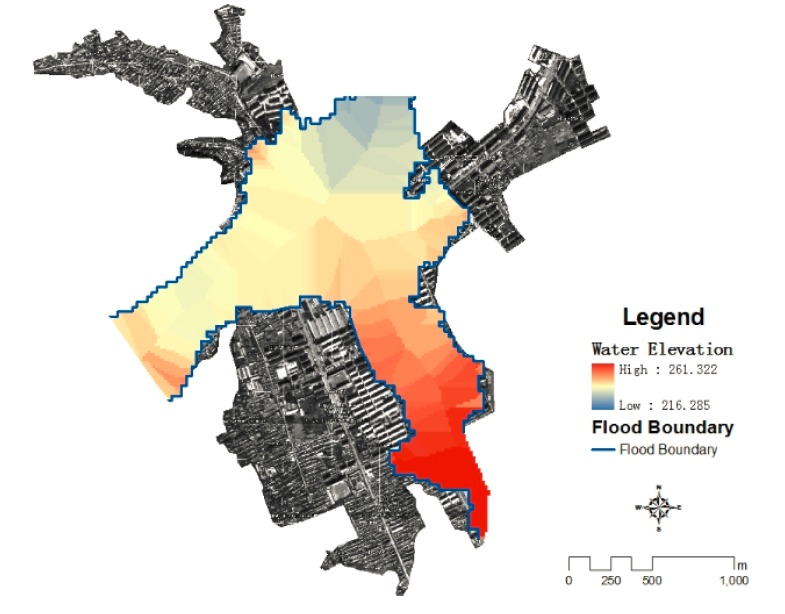
The result of flood surface elevation.

Flood depth could presented as water surface elevation minus land surface elevation. Water surface elevation could be extracted by spatial interpolation of flood boundary elevation. In this study, the flood boundary elevations were extracted by using the Mask tools of ArcGIS software and abnormal flood boundary elevation values should be excluded, using neighborhood statistical analysis of ArcGIS software, 90 m radius of the window and the mean function were chosen to smooth the elevation value of the submerged border. Voronoi Map is one of the boundary interpolation methods, it assumes that the spatial properties change occurs on the boundary, and the boundary within the value is determined by the nearest boundary value. Because this assumption is consistent with the actual situation, the Voronoi Map method is used to obtain the flood water surface elevation in this study. The elevation raster of flood submerged border was transferred into vector points using spatial analysis of ARCGIS software, and Thiessen polygons were generated by re-used statistical analysis module of ARCGIS software in order to obtain the flood water surface elevation (Figure 5).

**Figure 6:**
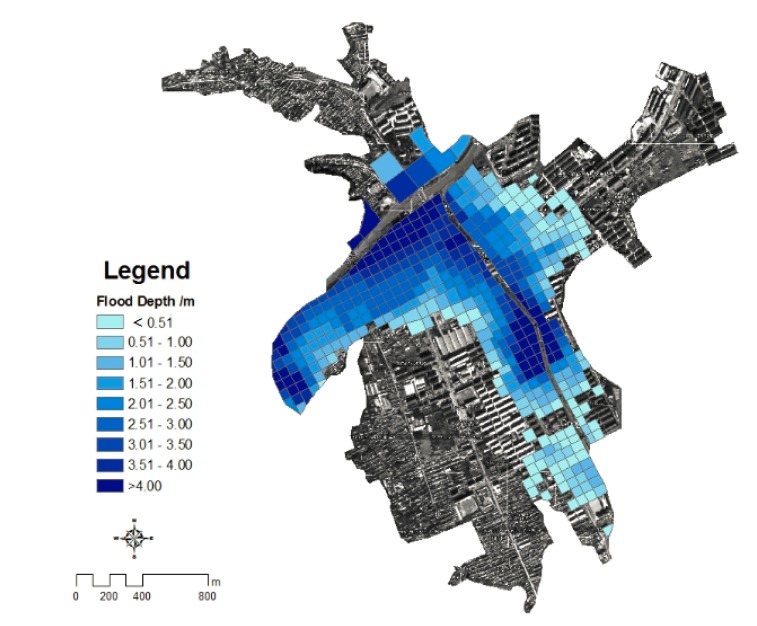
The depth of flood in the habitation of Kouqian Town.

DEM with a resolution of 5 m was generated by digitalized the topographical features elements such as contour lines and elevation points in 1:10,000 topographic map made by the State Bureau. Flood depth of each grids were calculated by water surface elevation subtracted the land surface elevation in each grid cell using ArcGIS software (Figure 6).

From Figure 6, nearly half of residential land in Kouqian Town had been submerged during the flood and the flood-inundated areas were mainly located on both sides of the rivers. The farther from the river, the flood depth is much shallower. The figure shows that the highest flood hazard area of Kouqian Town are mainly concentrated in the northwest, west and central regions, that is Old Street, the county hospital and Lianshan Bridge area, while the flood hazards in the southern and eastern regions are relatively small.

### 4.3. Housing Exposure Assessment of Kouqian Town

Exposure is often used to describe all persons and property that would be subjected to the threat of floods, such as personnel, buildings, crops and lifelines [7]. In this study, exposure presented as the number of external structure and internal property value of all types of residential housing. Residential area was extracted based on the 1:10,000 topographic map of the study area and using Google Earth image to amend it. It is based on the Google Earth image and field survey to classify the residential types, external structure and internal value of the housing.

**Figure 7:**
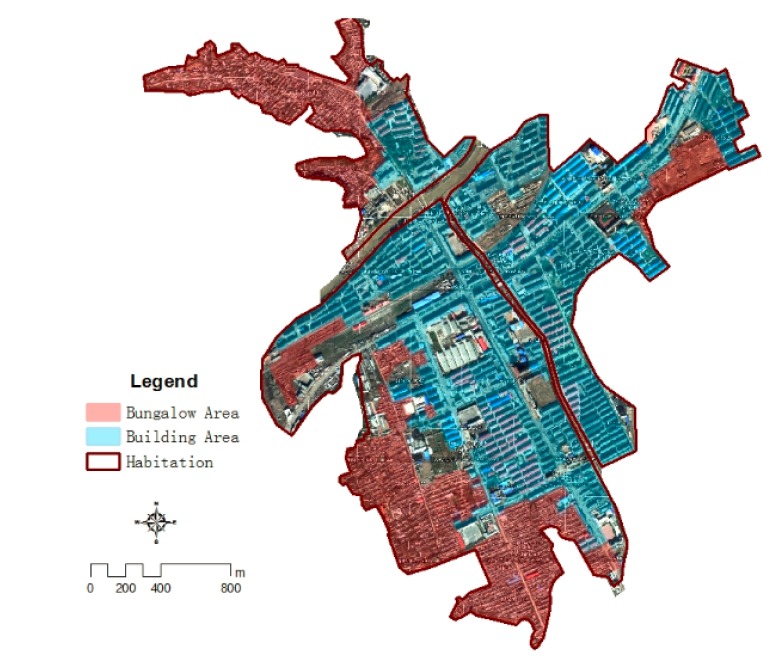
The classification of habitation in Kouqian Town.

There are mainly two types of residential housing and they are buildings and bungalows. Residential buildings and cottage housing can be roughly identified based on external morphological differences on Google Earth: the building body is wider and its shadow is longer, the spacing between buildings is also larger compared with bungalow. Field survey had been used to verify the classification results, and public buildings, such as schools, hospitals, administrative offices were verified during the fieldwork (Figure 7).

Residential property value (RP) in each grid cell is determined by the value of the external structure and internal value (IV) of the property according to the conceptual framework for assessing residential exposure. The value of external structure is calculated by the following function:

RP_ij_ = EH_ij_ + IV_ij_ (1)

**Figure 8:**
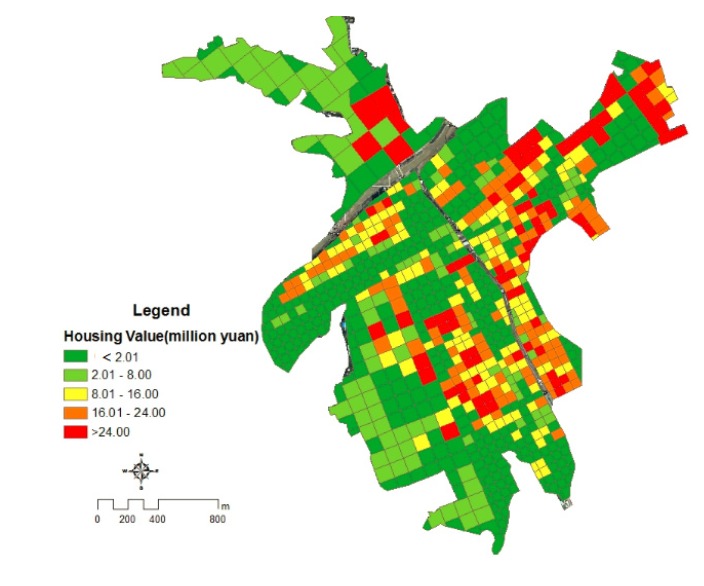
The distribution of housing property in Kouqian town.

EH_ij_ = HA_ij_ + PV_ij_ (2)

where EH_ij_ is the value of external housing value in grid cell (i, j), HA_ij_ is the housing area in grid cell (i, j), and PV_ij_ is the external housing value in per unit area. The internal property value is obtained by field investigation. Residential property value of each grid in Kouqian Town had been distributed by ArcGIS software (Figure 8).

From Figure 7, it can be seen that the differences of grid residential property are significant. The structures of the buildings’ external value and internal value property are much larger than the bungalows’, thus the value of grids in the building region is significantly greater than the value of grids in the cottage region. In addition, the total values of grids located in the dense region are much larger than that in the scattered region.

### 4.4. Vulnerability Assessment of Resident Housing in Kouqian Town

Vulnerability means the potential losses of bearing body caused by a natural hazard. When the hazards are the same, the larger the losses of a region are, the more vulnerability the region is [11]. Vulnerability is one of the important factors that constitute flood disaster risk, and it comprehensively reflects of the extent of the losses of the floods [12]. Through field surveys, residential damage levels and flood depth are closely related. Therefore, this study selected the depth of flood as the indicator to build flood depth—loss rate curves of all types of housing to represent the common vulnerability of all kinds of housing in Kouqian Town. 

#### 4.4.1. Vulnerability Evaluation Model of Housing of Flood

Residential losses rate of flood disaster refers to the ratio of loss subjected to flood cover the original value before the disaster [13]. Flood loss rate model of both external structure and internal property had been built based on the concept of flood loss rate. The vulnerability model of the external structure value is represented by:

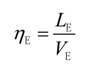
(3)


where *η*_E_ is the loss rate of external structure value; *L*_E_ is the flood loss of external structure; *V*_E_ is the external structure value of housing:

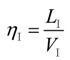
(4)


where *η*_I_ is the flood loss rate of internal structure value, *L*_I_ is the flood loss of internal structure, *V*_I_ is the internal structure value of housing.

#### 4.4.2. Depth-Loss Rate Curves of Housing in Kouqian Town

**Figure 9:**
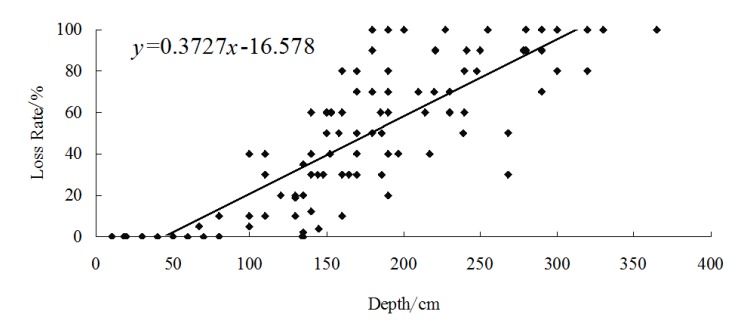
Vulnerability curve of the structure of bungalows in Kouqian Town.

**Figure 10:**
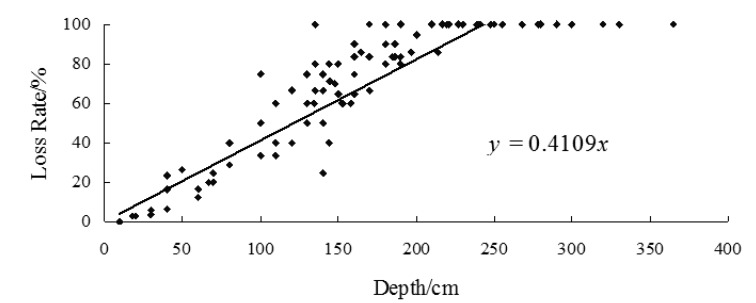
Vulnerability curve of the indoor property of bungalows in Kouqian Town.

A total number of 109 bungalows that suffered from the floods in the year of 2010 had been investigated during the fieldwork, using GPS to locate these 109 houses, and we used a questionnaire survey to obtain the basic information of all residences and the flood depths were measured. A linear regression model were used to fit flood depth to loss rate. The fitted line was the vulnerability curve (Figure 9 and Figure 10).

Depth of flood water and the rate of loss rate were entered into the SPSS software, and depth-loss rate regression equation of external structure value and internal property were built by using regression analysis methods. Through the comparative analysis of the correlation coefficient of each regression equation, linear regression model has the highest correlation coefficient. Depth-loss rate curves of external structure value and internal property are shown in Figure 9 and Figure 10, the coefficients are 0.6892 and 0.7208 at a 0.05 significance level. 

**Figure 11:**
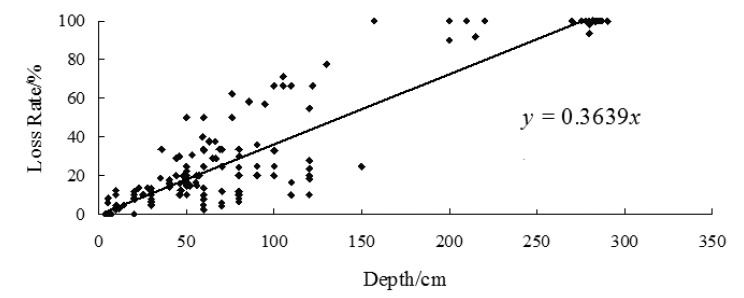
Vulnerability curve of the indoor property of buildings in Kouqian Town.

A total of 166 buildings suffered from the floods in 2010 had been investigated during the fieldwork. GPS were used to locate these 166 houses, and a questionnaire survey was used to obtain the basic information of all residences and the flood depths were measured. The flood depths of submerged buildings were between 0.1 and 4.65 m, and the structures of the buildings had not been destroyed. Thus, for buildings, only the depth-loss rate curve of the internal property had been established (Figure 11). The coefficient is 0.8792 at a 0.05 significance level.

#### 4.4.3. Grid Distribution of Housing Loss Rate in Kouqian Town

**Figure 12:**
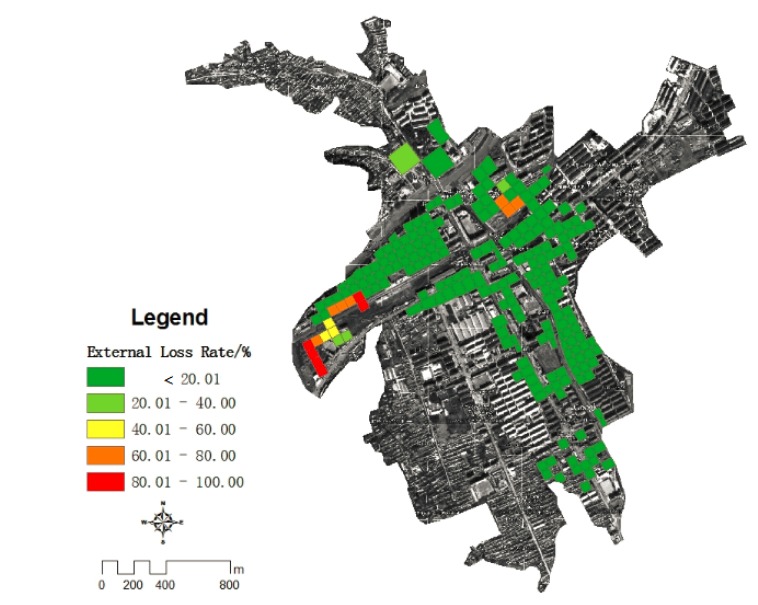
The loss ratio of the housing structures in Kouqian Town.

**Figure 13:**
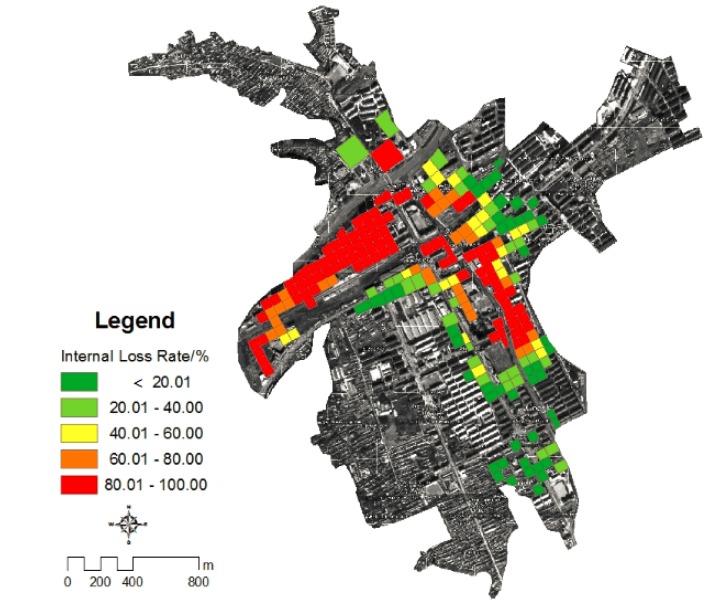
The loss ratio of the indoor property of houses in Kouqian Town.

Based on the results of flood depth in each grid cell and the vulnerability curves the loss rate of flood both external structure and internal property of all grid cells can be calculated (Figure 12 and Figure 13).

### 4.5. Flood Risk Evaluation of Resident Housing in Kouqian Town

**Figure 14:**
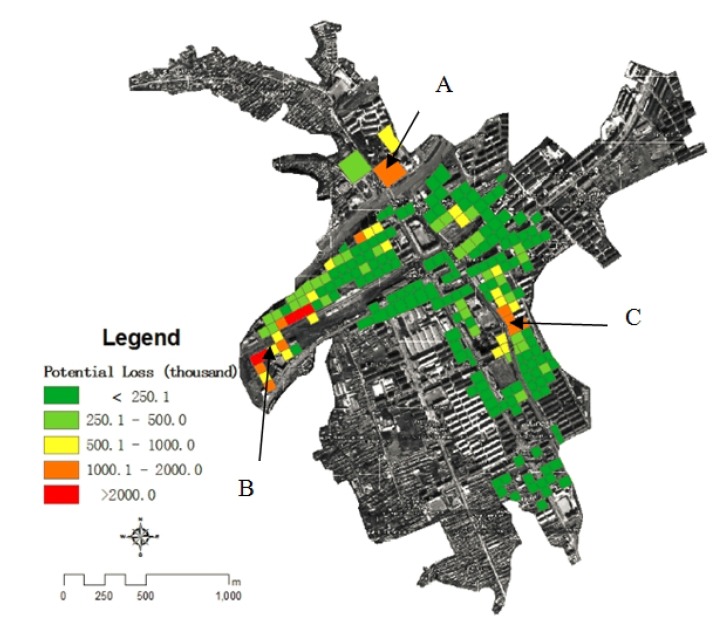
The potential loss of housing during the flood in Kouqian Town.

Flood disaster risk is described as the potential losses caused by flood. According to the concept and formation mechanism of the flood disaster risk, the flood potential losses are mainly affected by two factors: the total value of housing and the flood loss rate of housing. The value of housing includes external structural value and internal property value. Flood potential losses formula of housing is as follows:

*PD*(*i, j*) = *PVbs*(*i, j*) · *VVbs*(*i, j*) + *PVbc*(*i, j*) · *VVbc*(*i, j*) (5)

where for any grid (*i,j*) *PD* is the potential losses, *PVbs* is the property value of external structural, *VVbs* is the loss rate; *PVbc* is the property value of internal property, *VVbc* is the loss rate of it. By using the above formula, the potential flood losses in each grid cell was calculated (Figure 14).

Figure 14 shows that the high flood potential loss areas of Kouqian Town are mainly concentrated in three zones. By comparing with Figure 6, Figure 8, Figure 12 and Figure 13, it could be found that, for area A it has high exposure and high loss rate of indoor property, this is the reason of its high flood losses. For area B, the reason is the larger flood depth and high loss rate of housing structures. For area C, the reason is the larger flood depth, high exposure and large loss rate of indoor property, so flood risk is different for each area. To know the risk formation of each area has great value for disaster management and emergence rescue operations.

### 4.6. Verification of Potential Loss of Housing during the Flood in Kouqian Town

**Figure 15:**
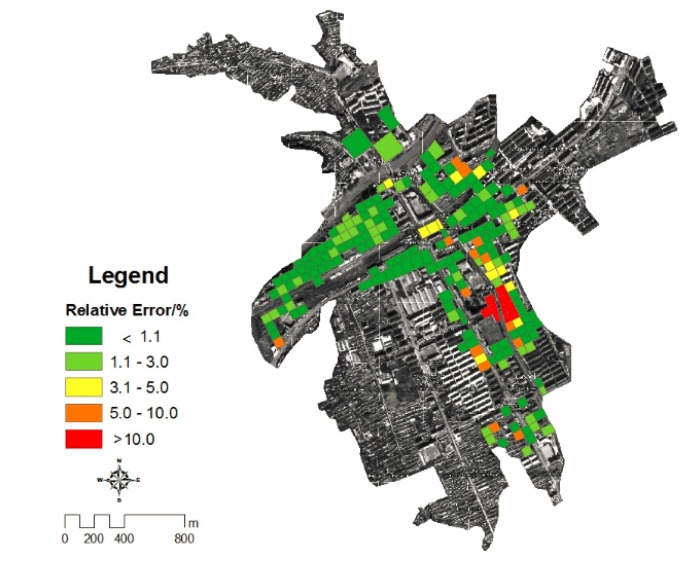
The verification of potential loss of housing during the flood in Kouqian Town.

The flood losses of housing in each grid that obtained by fieldwork were used to verify the accuracy of calculation in this study, and the verification result shown in Figure 15. From the figure, it can be seen that most of the grids’ actually flood losses are very close to the flood disaster risk assessment results with an average error of 2.88%, which can fully prove the reliability of the methods used in this study, so we can use these methods to assess the risk of other flood events, only using remote sensing images.

## 5. Conclusions

This study takes the flood that happened in Kouqian, Jilin Province on 28 July 2010 as an example to analyze the mechanism of formation of flood disasters. Taking grids as the assessment unit to assess the flood disaster housing losses from the three aspects of hazard, exposure and vulnerability, hazard was expressed as flood submerged area and depth, obtained by remote sensing images and DEM data. This study distributed the housing value, including housing structure and indoor property, into grids as the exposure factor. Based on the fieldwork of flood disaster losses the vulnerability of different kind of housing was assessed. Finally, this study built a fast assessment model for flood disaster losses.

(1) This study used remote sensing images, DEM data and GIS technology to extract flood submerged area and depth. The results coincide with the real fieldwork results. This method could replace fieldwork, so as to save human, physical and financial resources.
(2) For housing structures, buildings (multi-story) will not be destroyed under 4.5 m flood depth with a bit of losses; bungalows will not be destroyed under 1 m flood depth. The bungalows will be totally submerged if the flood depth is higher than 3 m and cause collapses. The higher the flood depth is, the larger the losses of housing structure are. When the flood depth is the same, the older the house is the larger the losses of the house are.
(3) For indoor properties, when the flood depth is higher than 2 m, all the indoor property will be destroyed. There also has a positive relationship between flood depth and indoor property losses.
(4) Based on the assessment of hazard, exposure and vulnerability of flood disaster, this study built s flood disaster losses fast assessment model. In different areas the disaster losses may be caused by different reasons, some may caused by large flood depth, some may caused by high housing values. The assessment result could be used in disaster management and emergence rescue.

As soon as a flood disaster happened, this assessment method can be used to assess the flood disaster losses from remote sensing images to get the flood submerged area and flood depth, then the depth-loss rate curves can be used to calculate the flood losses. It is rapid and can save human financial and material resources. In addition, from the perspective of other impact characteristics, flow rate and duration also impact the loss rate of housing; from a residential structure point of view, building foundation and construction materials also affect the flood loss rate of housing. These factors will be considered in a future study in order to improve the accuracy of the vulnerability curves and potential flood loss evaluation.
